# A static rooftop shading system for year-round thermal comfort and energy savings in hot climates

**DOI:** 10.1016/j.heliyon.2024.e31599

**Published:** 2024-05-31

**Authors:** Juana Isabel Méndez, Luis Ibarra, Pedro Ponce, Alan Meier, Arturo Molina

**Affiliations:** a Institute of Advanced Materials for Sustainable Manufacturing, Tecnologico de Monterrey, Puente 222, Tlalpan, 14380, Mexico City, Mexico; b Energy and Efficiency Institute, University of California, Davis, 1605 Tilia St 100, Davis, 95616, CA, USA

**Keywords:** Passive strategies, Indoor temperature, Rooftop strategies, Adaptive thermal comfort, Energy simulations

## Abstract

In sun-drenched regions, balancing solar exposure for thermal comfort and minimization of cooling energy presents a key challenge. While passive shading mitigates summer heat gain, it also hinders winter solar benefits, a problem that is echoed by active systems such as photovoltaic panels. Existing adaptive solutions, adjusting to seasonal sun angles, offer flexibility, but introduce complexity, maintenance demands, and potentially higher costs. This study introduces a novel static roof shading system that addresses this knowledge gap. It effectively blocks summer sunrays and allows winter insolation without seasonal adjustments or associated mechanisms. This “install and forget” approach promises improved thermal comfort and reduced energy consumption in hot climates. Rigorous energy simulations in nine different Mexican locations, using a 69 m^2^ household model, reveal significant temperature improvements. The system delivers up to 30.38% (above 5 MWh) equivalent annual reduction in HVAC usage and 71.3% relative increase in thermal comfort during hot periods. This sustainable solution offers valuable information to address indoor thermal challenges in high-temperature regions, contributing to the advancement of sustainable building technologies.

## Highlights

• Introduced a novel fixed rooftop shading system for year-round temperature modulation.
• Simulations in nine Mexican cities validated the system using hourly data for a year.
• Resulted in up to 71.3% thermal comfort in non-cooled households.
• Achieved up to 30.38% annual cooling energy reduction in households using HVAC.

## Graphical abstract

**Figure:**
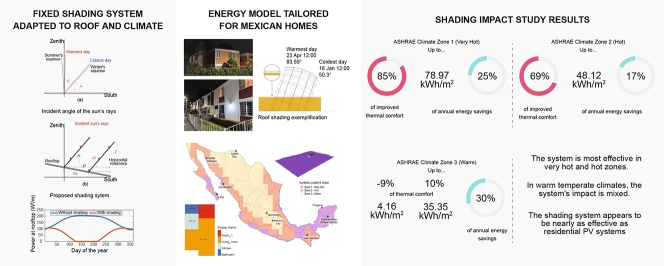

## 1 Introduction

Traditionally, the control of indoor temperatures in buildings has been based on active energy-intensive technological solutions [1], [2]. In recent years, researchers and the construction industry have increasingly sought to optimize resource usage and retrofit buildings. For example, Ascione et al. [3] focused on optimizing solar energy utilization in retrofitted buildings within an Italian neighborhood by integrating photovoltaic (PV) systems, HVAC systems, and building envelopes. They analyze a comprehensive range of energy efficiency measures and the effects of the Italian “Superbonus 110%” public grant policy, highlighting how financial incentives can drastically influence the cost effectiveness of different energy retrofits and promote PV systems to reduce environmental impact.

Similarly, Calama-González et al. [4] proposed optimal retrofit solutions for the Mediterranean social housing stock, focusing on a balance between thermal comfort and cost. Their work shows that medium-cost strategies can significantly reduce discomfort hours compared to high-cost options, which is crucial for sustainable practices in residential areas. Furthermore, Manjarres et al. [5] contribute to this field by proposing multi-objective algorithms for district energy retrofitting, focusing on historical city centers. Using algorithms such as MOHS and NSGA-II, their research aims to achieve energy and economic goals simultaneously, offering a dynamic and effective way to plan urban energy renovations while maintaining economic feasibility.

Beyond the said interest and efforts to find an effective allocation of resources, other proposals have gone a step further and embraced passive alternatives for thermal control [6], [7]. This shift emphasizes *passive architecture* and *bioclimatics*, aiming to optimize building performance while minimizing energy consumption by harnessing local environmental conditions and construction practices [8]: use the least energy to meet temperature, lighting, humidity and airflow requirements, among others.

As climate change increases temperature, it is predicted that heating energy needs will decrease while cooling demands will increase [9]; also, the cooling potential of ventilation will decrease over time [6]. This problem is exacerbated in regions with low-cost housing or cultural disregard for insulation practices [10], leading to the reliance on energy-intensive solutions, such as air conditioning. In Mexico,^1^ where the temperate weather is dominant [10], this pattern results in seasonal energy spikes of approximately 30% during summer, mainly due to the use of air conditioning [11]. This not only increases greenhouse gas emissions, but also poses public health risks and economic concerns, with the government subsidizing a substantial portion of the electricity costs for residential air conditioning: in an estimate of 2018, 60% of the electricity costs of HVAC in the residential sector were paid by the government [11].

Passive cooling, which is based on natural or physical properties rather than electricity, emerges as a cost-effective and eco-friendly approach to residential cooling [12]. Critical factors include window size and placement, especially window-to-wall and window-to-floor ratios [13], [14], [9]. Innovative techniques, such as phase-change materials for wall insulation, have been proposed [13]. However, retrofitting existing buildings with such strategies can be challenging.

Therefore, features such as overhangs, windows, awnings, vegetation, and textured surfaces are recommended to regulate indoor temperatures by reducing solar exposure in facades and windows [15]. These measures have shown promise, achieving cooling load reductions ranging from 27.5% to 64.5% in various contexts [16], [6], and up to a decrease of 74% in cases of overheating [17]. However, it is essential to note that *shading* is not universally applicable and requires case-specific analyzes [18], [17]. For example, in Saudi Arabia, the thermal mass outperformed the wall shading [19]. Some propose scheduled or adaptive shading to maximize benefits [16], [20], [9], either as a standalone strategy or in combination with other solutions [13].

Adaptive shading becomes essential when there is a simultaneous need to utilize sunlight for indoor heating or to generate photovoltaic power. Fixed shading, as noted by certain authors, can inadvertently lead to adverse effects such as increased heating requirements and higher energy consumption for lighting [17], [19]. To achieve this desired adaptability, there are two primary approaches: user intervention and automated mechanisms. Numerous studies have explored the optimization of shading strategies by implementing methods such as seasonal operating modes or pre-defined shading schedules to maximize their advantages [20], [9]. However, it is important to acknowledge that the incorporation of automated shading systems often comes with increased costs, which may present practical limitations in their widespread application [21].

Although many contributions in the field of specialized shading techniques focus primarily on facades and windows [22], [16], [23], the potential of such strategies for rooftop applications has remained largely overlooked. In the context of Mexico, it is customary to construct houses with flat rooftops located close to each other, regardless of orientation. This distinctive architectural practice produces specific conditions that hinder the application of conventional shading solutions. Furthermore, it should be noted that Mexican homes often lack professional insulation [10], which contributes to increased energy consumption and discomfort for residents.

To the best of the authors' knowledge, only one documented endeavor has faced a comparable challenge. In a recent investigation conducted by García-Solórzano et al. [11], they introduced an intricate wooden meshed canopy designed for rooftop deployment, allowing adjustable shading by manipulating both its aperture size and elevation. Through substantial experimentation, the most suitable configuration for a particular geographic location was determined during different seasons of the year. The findings revealed which parameters and adjustments were effective in enhancing operational temperatures for specific seasonal conditions.

Unlike the works cited, our study presents a static passive shading system tailored for rooftops, notable for its simpler composition and minimal adjustment requirements. The primary objective is to block sunlight during the hot seasons while allowing its ingress during colder periods (without adaptive mechanisms). Unlike the proposal of García-Solórzano et al. [11], the present proposal operates effectively throughout the year with a single initial configuration. This eliminates the need for seasonal adaptations and associated complexities. Furthermore, the analysis delves into the concept of thermal comfort (counting for humidity and air velocity alongside temperature) and the system's potential for energy savings, providing a more comprehensive assessment of its impact on building performance. Finally, the proposed system uses historical data that are readily available for most regions, facilitating broader applicability and scalability without requiring complex on-site investigations.

This study is distributed as follows. Section 2 outlines the shade design and adjustment, details the household design and the locations considered to perform the tests, and introduces the energy model used. Section 3 shows the parameterization of the shading system for each location and details the study results. Section 4 highlights the effectiveness and limitations of the proposal, and Section 5 highlights the most important results, provides some conclusive remarks, and states the proposed future work.

## 2 Proposal

This study outlines a comprehensive three-step approach. Firstly, we introduce the shading system alongside the mathematical equations that facilitate its placement over various rooftop inclinations and considering historical temperature data. This initial step is presented in a generic context to underscore the versatility of the system and its potential applicability across diverse geographical locations.

Secondly, we present the building energy model, meticulously tailored to reflect the distinctive characteristics of typical Mexican households. This comprehensive model incorporates parameters such as floor area, construction materials, air conditioning usage patterns, spatial disposition relative to neighboring structures, and environmental conditions of households' location during the year 2021. These conditions include key factors such as solar positioning, wind velocity, and ambient air temperature.

In the ensuing phase, the energy models are executed with and without the shading system to assess its impact on rooftop surface temperature, total air conditioning consumption, and indoor temperature variations throughout the same year and under the same simulation directives. Finally, a series of 36 energy simulations (two per location to enable comparisons), one considering just natural ventilation and the other considering HVAC, are conducted across various geographic locations to evaluate the shading system's effectiveness rigorously, discern the pivotal variables influencing outcomes, and delineate quantitative performance metrics.

### 2.1 Description of the shading system

**Figure 1:**
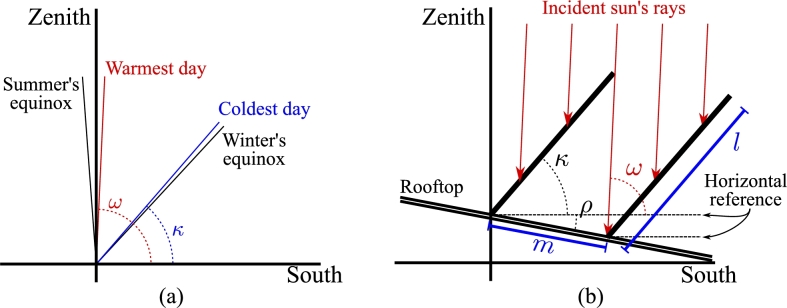
Cross-sectional view of (a) the incident angle of the sun's rays depending on the time of year, and (b) the proposed shading system over a roof, with adjustment marks. A northern hemisphere latitude was considered.

The proposed shading system comprises rigid, flat, opaque shades, and a support system determining their inclination and separation (see Fig. 1). All shades are inclined to the same degree and are expected to become coaxial with the sun's rays at some desired time of the year, enabling the rooftop full insolation. On the other hand, they will obstruct the sun's rays fully above a calculated incidence angle, making all sun's light land only over the shading system and depriving the rooftop of insolation.

The shading system is intended to obstruct insolation fully during hot weather but to allow the sun's energy to reach the rooftop during the cold season. By doing this, it would be possible to reduce the amount of heat that contributes to the overall indoor temperature when the air temperature is already high, but avoiding isolation when radiating heat could be helpful. In addition, it is desired that the system operates in an “install and forget” manner, i.e., to be adjusted only once without further variations.

#### 2.1.1 Adjustment of the shades

Two parameters are necessary to adjust the proposed system, both shared among all shades. Fig. 1(b) shows a cross-sectional view of the shading system where the inclination *κ* (with respect to the horizon) and the separation *m* (between two shades) are visible. Both parameters can be adjusted using the latitude, the rooftop inclination *ρ* (with respect to the horizon), and historical data on the temperature in the location of interest.

Since the system is intended to let light through during cold days, it is necessary to analyze historical weather data and find the sun's elevation around the coldest recorded day *κ*. In the northern hemisphere, it should be close to the sun's elevation during the winter equinox. However, the system aims to block sunlight during the hottest days. Then, the specific elevation of the sun can be analyzed from historical data to obtain the associated incident angle *ω*, which should be close to the sun's elevation during the summer equinox. Fig. 1(a) shows an example considering a latitude of $19.4326^{\circ }$ N, using historical data from Mexico City, Mexico.

Then, the shades' inclination must be *κ*, whereas the separation among shades can be easily computed incorporating *ω* and *ρ*, i.e.,

(1) $$m=l\left(\frac{\cos(\kappa )-\sin(\kappa +\rho )}{\tan(\omega )}\right)$$

where *l* is the shades' longitude. Equation (1) produces the necessary distance between the shades to ensure the obstruction of sunlight for an incident angle *θ* beyond *ω*. Throughout the year, *θ* will vary, and the system will only shade a portion of the roof. Such a portion between two shades, *s*, can be computed with

(2) $$s=l\left(\frac{\cos(\kappa )-\sin(\kappa +\rho )}{\tan(\theta )}\right).$$

Clearly, the alight proportion between two shades is a=m−s, and p=a/m outputs the sunlit proportion of the roof.

#### 2.1.2 Adjustment example

Insolation data were obtained from the NASA - GISS webpage [24] for 2022 at $19.4326^{\circ }$N - $99.1332^{\circ }$W, corresponding to Mexico City, Mexico. Such data consider insolation at the top of the atmosphere and the site states that about 57% reaches the Earth's surface. The data obtained also include the sun's zenith angle at noon [^∘^], the daily average sunlight [W/m^2^], and the *sunlight-weighed cosine of the zenith angle*, a factor that weighs the received power depending on the incident angle and other deductions.

The air temperatures from 2022 were also recovered from a weather station at the local airport. The coldest day was found to be 14 January, corresponding to a sun angle of $\kappa =49.09^{\circ }$, and the hottest day was 7 May, with a sun angle of $\omega =87.51^{\circ }$. Most houses in Mexico City are built with a flat rooftop, i.e., $\rho =0^{\circ }$.

Therefore, the distance between the shades was calculated as m=0.622l, while the alight proportion *p* was calculated for every day, considering the daily sun's angle. Finally, the weighed power, considering the angle of the zenith and the average losses of the atmosphere, was calculated and multiplied by *p* to compare the incident power on the roof with and without the shading system.

**Figure 2:**
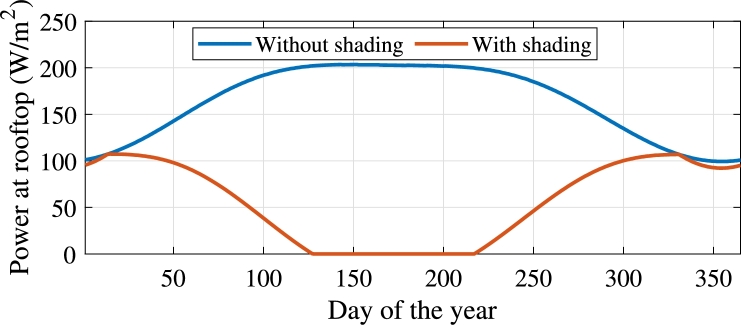
Average received power of a hypothetical rooftop in Mexico City.

The results are shown in Fig. 2. It can be seen that maximum and minimum insolation correspond to summer and winter, respectively, which are the hot and cold seasons for the northern hemisphere. In its turn, the shading system was adjusted to fully block sunlight on 7 May (day 127), when the rooftop power reaches zero. However, sunlight is allowed through during the cold season, showing p=1 exactly on 14 January (day 14) as designed.

To automate the shade adjustment process, Grasshopper v1.0.0007 was used. Thus, the calculations described above were used following these steps for every location considered:

1. Download the weather file from Climate One Building [25].
2. Obtain the hottest day at 12:00 p.m.
3. Obtain the coldest day at 12:00 p.m.
4. Calculate the inclination of the shades.
5. Calculate the separation of shades.
6. Automatically generate the shades within the software simulation.

### 2.2 Locations considered

**Figure 3:**
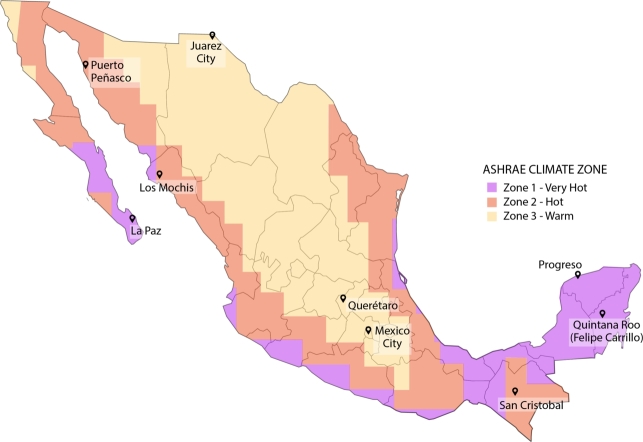
Mexico with ASHRAE map [26].

In terms of the context of the ASHRAE climate zones, Mexico predominantly falls under three distinct climate classifications: “very hot” (zone 1), “hot” (zone 2), and “warm” (zone 3). Fig. 3 illustrates the ASHRAE climate classification specific to Mexico. Similarly, the National Survey of Energy Consumption in Private Homes (ENCEVI) [10] classifies Mexico into three climatic regions: “extremely hot,” “temperate,” and “tropical”. It is pertinent to highlight these distinctions because, for example, while ASHRAE designates Juarez City as a “warm” zone, the Mexican classification categorizes it as an “extremely hot” region.

Numerous techniques and studies to improve building thermal control often rely on tests conducted in different climate regions. However, as shown, these are not exactly coincident and may lead to considerable variability among specific locations. Moreover, the current proposal hinges on adjusting the shading system based on outdoor temperature. Therefore, it is necessary to account for both the climate zone classification, which impacts the construction templates integrated into the simulation, and the stochastic nature of temperature fluctuations. The Mexican states that fall within these aforementioned climatic regions are as follows (bold-faced states are the focus of this study):

• Extremely hot region: Baja California, **Baja California Sur**, **Sonora**, **Chihuahua**, Coahuila, Nuevo León, Tamaulipas, **Sinaloa** and Durango.
• Temperate region: Nayarit, Jalisco, Colima, Michoacán, Zacatecas, Aguascalientes, San Luis Potosí, Guanajuato, **Querétaro**, Hidalgo, Estado de México, **Mexico City**, Morelos, Tlaxcala and Puebla.
• Tropical region: Guerrero, Oaxaca, Veracruz, **Chiapas**, Tabasco, Campeche, **Yucatán** and **Quintana Roo**.

#### 2.2.1 Characteristics of the household

The 2018 ENCEVI included a survey of 32,047 households. The findings of this survey underscore the prevalent lack of thermal insulation in a significant majority of these households. Specifically, in the “extremely hot” region, a striking 85% of households lack thermal insulation, while this figure increases to 98.5% in the “temperate” and “tropical” regions.

In addition, the survey reveals distinct disparities in the adoption of air conditioning systems. In the extremely hot region, approximately 48.1% of households employ them, while in the temperate region, this figure drops drastically to just 1.3%, and in the tropical region it is 12.4%. During the winter months, 20.4% of households in extremely hot regions use heating systems, while in the temperate region, usage is considerably lower at 2.5%, and in the tropical region it is 1.6%.

**Figure 4:**
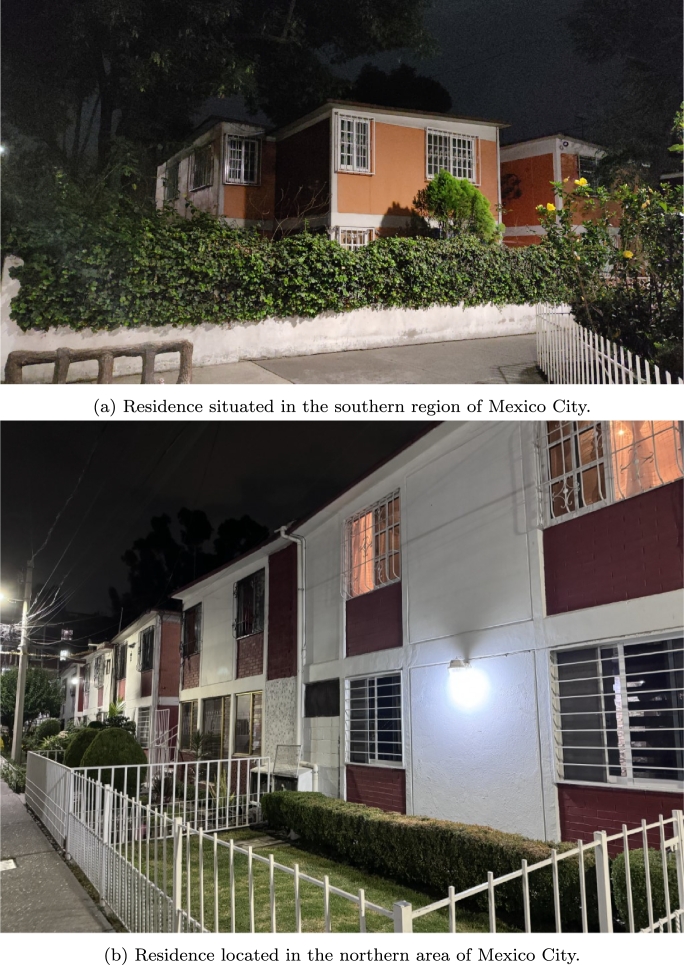
Typical Mexican residential complex.

Regarding the area of households, the National Institute of Statistics and Geography (INEGI) reported that homes ranging from 56 to 100 m^2^ of construction represent the highest proportion of homes in the country, with 41.3% [27]. Fig. 4 depicts how Mexican houses are typically layed out; for example, Fig. 4a illustrates a household in the southern area of Mexico City, whereas Fig. 4b depicts one in the northern region of the city.

**Figure 5:**
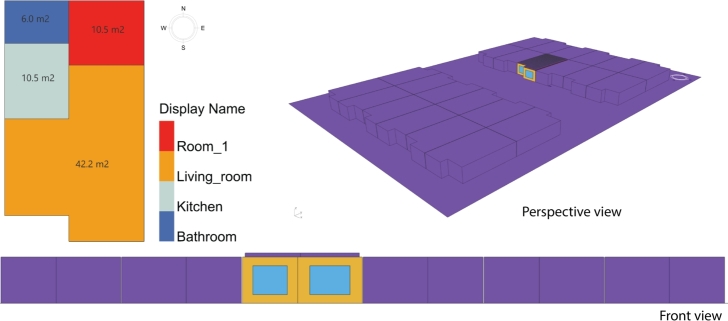
General distribution of the household with its context.

The building under consideration is designed as a typical one-level Mexican house in a residential complex, surrounded by adjacent households (terraced houses). Fig. 4 shows a two-story building comprising two living units (duplex); however, for the purposes of this investigation, only a single level (or the upper level) was modeled, considering the shading provided by the adjacent houses. However, surrounding vegetation was not included in the model, which was calibrated against an existing upper-level household. Fig. 5 shows the general distribution and shows a 3D model of this typical 69 m^2^ household. The model considers four zones:

• Room 1: 10.5 m^2^
• Living room: 42.2 m^2^
• Kitchen: 10.5 m^2^
• Bathroom: 6 m^2^

The energy simulations were performed considering the same distribution but at different locations. The windows were located in the front part of the household. As is commented on in the sequel, the above details were incorporated into the performed simulations and were considered during the results' analysis.

### 2.3 Tests considered

Although the proposed shading system could adapt to satisfy the inclination corresponding to a wide variety of locations, it is expected that the maximum benefit is obtained in places with hot air's temperature and long periods of sun's irradiation. To validate this assumption, an hourly temperature examination must be performed in each location to determine any relationship between outdoor and indoor temperatures. Historical data from 2022 were used.

The proposed system can provide benefits in two main ways. Firstly, it could improve indoor temperature during the hot season, indirectly improving comfort. To this end, a set of tests was run to acquire indoor temperatures with and without the shading system, and their difference was computed. Secondly, the proposed system could reduce the energy needed for cooling purposes. A second set of tests considered the same household but equipped with heating and HVAC systems, set to ensure indoor temperatures between $21.7\;{}^{\circ }$C and $24.4\;{}^{\circ }$C. Then, the cooling and heating required energies were computed and compared.

The indoor temperature was captured per room and the overall indoor temperature was calculated as a weighed mean given in terms of the area that each room takes from the total area of the household. Comfort was calculated directly in the simulation software following the ASHRAE Standard 55. The cooling and heating systems were later added to all rooms except the bathroom and were considered to be *ideal*: able to manage any required cooling or heating load.

The simulation considered the variations of the home given by its inhabitants such as the closing and opening of doors and windows. Also, clouds would partially block the sun's rays, adding variability to the considered simulation. In addition, it is clear that daily temperatures will vary periodically since days are hotter than nights. Therefore, the resulting data must be decomposed to eliminate daily seasonality and overall noise. The resulting “clean” trends were achieved using R in all resulting temperature time series. In their turn, consumed energies were also processed as previously described only for visualization purposes, but their totals were computed summing every hourly value.

### 2.4 Creation of the energy model

EnergyPlus™ 23.1.0 [28] is a building energy simulation software developed by the US Department of Energy. It models and analyzes the energy performance of buildings, including their heating, cooling, ventilation, lighting, equipment, and other systems. EnergyPlus™ [28] is a key component of LadybugTools v1.7.0 [29], a plugin for Rhinoceros 3D v7 [30] and Grasshopper v1.0.0007 (included within Rhinoceros 7 software). Thus, LadybugTools v1.7.0 [29] uses EnergyPlus™ 23.1.0 [28] as its simulation engine for building energy and environmental analysis and is widely employed in research and industrial fields [31].

In addition to constructing the typical Mexican household model described, incorporating the shading system can be automated depending on the preloaded templates mentioned. In addition, these preloaded templates automatically consider the HVAC system automatically; EnergyPlus™ 23.1.0 [28] calculates the necessary heating and cooling demands to uphold specific thermal conditions inside the building. It also calculates secondary HVAC system and coil loads, and estimates the primary plant equipment energy consumption. This comprehensive simulation provides essential details to ensure the simulated results closely mirror real-world building performance [32].

#### 2.4.1 Thermal comfort

The concept of adaptive thermal comfort, originally introduced by Nicol and Humphreys [33], has significantly influenced indoor environmental design. This approach considers the dynamic relationship between indoor temperature and outdoor conditions, particularly the exponentially weighted running mean of outdoor temperature. At its core, adaptive thermal comfort recognizes that individuals respond to environmental changes to maintain their comfort [33], [34].

Parkinson et al. [35] and de Dear et al. [36] explored applying adaptive thermal comfort principles in different types of buildings. Although naturally ventilated buildings have readily embraced the adaptive model, mixed-mode structures face challenges reconciling conflicting guidance from heat-balance models and adaptive comfort principles. In contrast, air-conditioned buildings have slowly adopted adaptive strategies, prompting practitioners to investigate potential benefits and collect data to improve the control logic of the HVAC system [35], [36].

With its occupants' greater autonomy over their indoor environment, the residential sector has proven to be an ideal fit for adaptive thermal comfort principles. Residents can easily adjust the insulation of their clothing and tolerate a wider range of indoor temperatures, particularly during the transition seasons [37]. An essential feature of adaptive comfort models is their reliance on outdoor temperature, which often eliminates the need to consider other factors such as humidity or air movement. Such simplicity improves the practicality of the model [33].

ASHRAE Standard 55 considers the adaptive thermal comfort model by acknowledging that individuals can adapt to different temperature ranges over time, considering that they have different comfort expectations during different seasons [38], [39], [36]. The standard considers two types of acceptability: 90% and 80%. This percentage of occupants find indoor thermal conditions comfortable without additional adjustment. For residential sector homes, such as single-family homes or typical apartment buildings, targeting 80% acceptability is common and often sufficient [40].

## 3 Results

This section reports the results at each step of the study, beginning with parameterization of the shading system using Grasshopper v1.0.0007 based on reference equations. It explains how the shading system was designed for three distinct ASHRAE climate zones in nine selected Mexican cities. The following housing characteristics are described: a Mexican residential complex, outdoor temperatures, and energy model characteristics. Finally, the analysis of the energy simulation by zone is detailed.

### 3.1 Shading system

In light of the previously discussed climatic variability and to pinpoint the pertinent factors that contribute to the desired results, a total of nine cities were chosen. This selection encompassed three cities per climate zone:

• Zone 1 (very hot) - (i) La Paz, Baja California Sur; (ii) Progreso, Yucatan; (iii) Felipe Carrillo Puerto, Quintana Roo.
• Zone 2 (hot) - (iv) Los Mochis, Sinaloa; (v) Puerto Peñasco, Sonora; (vi) San Cristobal, Chiapas.
• Zone 3 (warm) - (vii) Juarez City, Chihuahua; (viii) Mexico City; (ix) Queretaro City, Queretaro.

**Figure 6:**
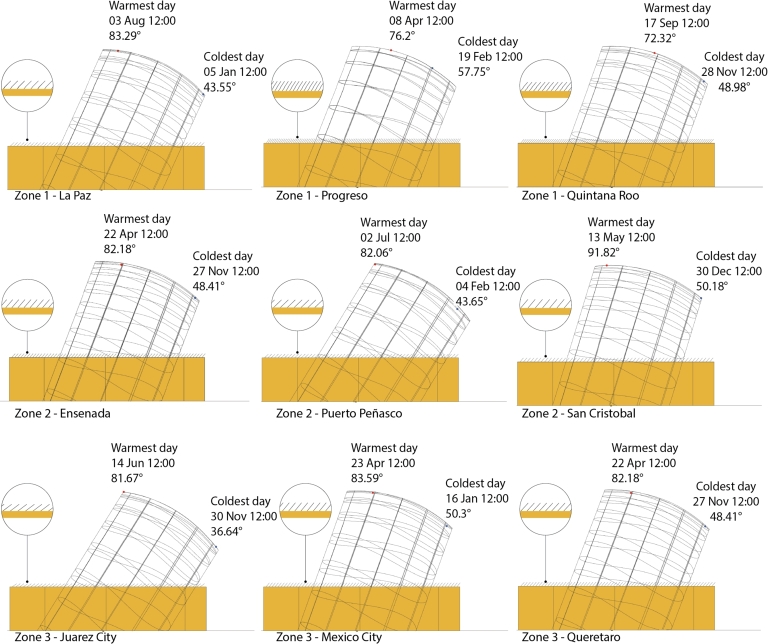
West View: roof shades distribution in each location based on the warmest and coldest days and as a result of (2).

Data were obtained from the EPW map collected by LadybugTools v1.7.0 [29] from different weather stations [41] (see Table 1). In addition, the shading system was parameterized (*ω*, *κ*, and *s*) for each location. Finally, a year round simulation was run with the location-specific conditions mentioned. The process was automated using Grasshopper v1.0.0007: one EPW file was loaded, the shades were automatically distributed and oriented over the roof, and the simulation was run. Therefore, as a result, Fig. 6 shows the adjustments of the shading system at each location to appreciate how the shades are distributed differently.

**Table 1:** Shading system considering *l* = 0.30 m for each selected location.

City	Ref.	Lat	Long	Inclination	Incidence	Separation
		(^∘^N)	(^∘^W)	*κ* (^∘^)	*ω* (^∘^)	*s* (m)
**Los Mochis**	[42]	25.68	-109.08	48.41	82.18	0.17
**Puerto Peñasco**	[43]	31.3	-113.5	43.65	82.06	0.19
**San Cristobal**	[44]	16.75	-92.63	50.18	91.82	0.20
**La Paz**	[45]	24.17	-110.3	43.55	83.29	0.19
**Progreso**	[46]	21.28	-89.65	57.75	76.2	0.10
**F.C. Puerto**	[47]	19.7	-87.9	48.98	72.32	0.12
**Juarez City**	[48]	31.64	-106.43	36.64	81.67	0.21
**Mexico City**	[49]	19.4	-99.18	50.3	83.59	0.17
**Queretaro City**	[50]	20.56	-100.37	48.41	82.18	0.17

### 3.2 Outdoor temperature

An examination was conducted on hourly temperature datasets from EPW files of each location to enhance the characterization of temperature patterns within distinct Mexican cities. Table 2 shows the summary statistics of outdoor temperature.

**Table 2:** Outdoor temperature summary statistics.

City	Min	Q1	Mean	Median	Q3	Max	HDH	CDH
	(^∘^C)	(^∘^C)	(^∘^C)	(^∘^C)	(^∘^C)	(^∘^C)	(hours)	(hours)
**Progreso**	17.9	24.2	26.2	26.3	26.3	34.7	4	2885
**Los Mochis**	9.0	20.0	24.6	25.0	29.0	39.0	167	2464
**F.C. Puerto**	12.8	22.4	25.4	25.2	28.8	38.4	62	2648
**La Paz**	6.3	20.1	25.0	24.8	24.8	43.1	128	2573
**Puerto Peñasco**	0.9	17.7	22.9	22.8	28.9	45.7	338	2031
**Juarez City**	-11.5	11.2	18.8	20.0	26.3	49.0	1417	1616
**Queretaro City**	1.0	14.0	18.0	18.0	22.7	35.8	967	854
**Mexico City**	2.7	13.0	16.3	15.9	19.9	30.3	1158	427
**San Cristobal**	5.4	13.3	15.8	15.5	18.6	26.6	1171	258

• Min (minimum temperature): lowest temperature record. Juarez City [48] has the lowest minimum temperature ($-11.5\;{}^{\circ }$C), indicating that it can experience very cold winter conditions. In contrast, Progreso [46] has the highest minimum temperature ($17.9\;{}^{\circ }$C).
• Max (maximum temperature): highest temperature record. Juarez City [48] also has the highest maximum temperature ($49\;{}^{\circ }$C), indicating extreme heat throughout the year. On the contrary, San Cristobal [44] has the lowest maximum temperature (26.6 °C).
• Mean (temperature): average temperature. Progreso [46] has the highest mean temperature ($26.2\;{}^{\circ }$C), while San Cristobal [44] has the lowest ($15.8\;{}^{\circ }$C).
• Median (temperature): median temperature. Progreso [46] and F.C. Puerto [47] have relatively high median temperatures ($26.3\;{}^{\circ }$C and $25.2\;{}^{\circ }$C, respectively), indicating consistent moderate temperatures. San Cristobal [44] has the lowest median temperature ($15.5\;{}^{\circ }$C).
• Q1 (first quartile): temperature in the 25% percentile. Together with Q3 (below), one can compute the interquartilic range, a dispersion metric, using IQR=Q3−Q1.
• Q3 (third quartile): temperature in the 75% percentile. Juarez City exhibits the highest temperature IQR ($15.1\;{}^{\circ }$C) whereas Progreso the lowest ($2.1\;{}^{\circ }$C).
• HDH (Heating Degree Hours): number of hours that require heating to keep a temperature above $18.3\;{}^{\circ }$C. Cities with higher values, such as Juarez City [48], experience colder temperatures and may require more heating to keep residential spaces comfortable. Progreso [46] has the lowest HDH (4), indicating minimal heating needs. Juarez City has the highest HDH (1417), suggesting significant heating demands due to cold temperatures.
• CDH (Cooling Degree Hours): number of hours that require cooling to keep a temperature below $18.3\;{}^{\circ }$C. Cities with higher CDH values, such as Progreso [46] and F.C. Puerto [47], have warmer temperatures and may require more cooling to maintain indoor comfort. This can lead to increased electricity usage for air conditioning; Progreso has the highest CDH (2885), indicating a higher demand for cooling.

**Figure 7:**
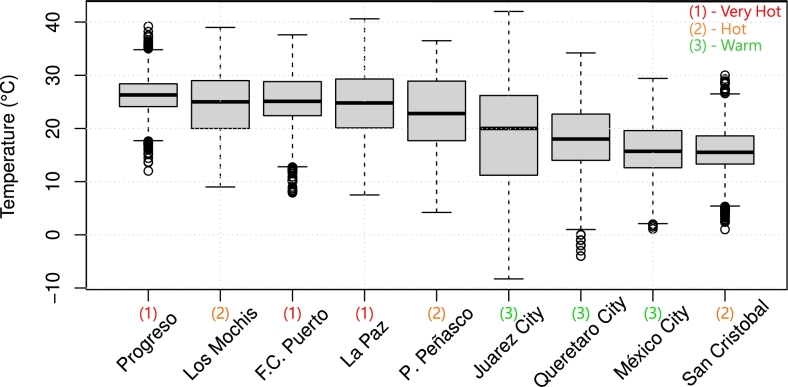
Ambient temperature boxplot of 2022 for each location considered. Locations were ordered based on median temperature. It should be noted that classification through climate zones may not adequately represent year-round temperatures.

The findings derived from this analysis are visually presented in Fig. 7. It can be seen that places located in the Very Hot (Zone 1) climate zone, such as La Paz, Progreso, and Felipe Carillo Puerto, or places like Los Mochis, Puerto Peñasco, and Juarez City, require cooling systems extensively. Therefore, building designs in these zones need energy-efficient strategies that prioritize effective cooling or provide a cooler space due to passive systems to reduce the demand for cooling energy.

San Cristobal, located in a tropical Mexican region or ASHRAE climate zone 2 (Hot), Mexico City and Queretaro City (located in ASHRAE climate zone 3 - warm), have lower CDH values, indicating a lower cooling requirement. However, passive cooling strategies may be needed to reduce cooling demands, especially in urban areas with higher heat absorption due to buildings and infrastructure.

Locations such as Juarez City, San Cristobal, and Mexico City have higher HDH values, indicating the need for heating systems during colder periods and passive designs focused on reducing heating energy consumption.

### 3.3 Creation of the energy model

Although ENCEVI classifies the different regions in Mexico better, the energy simulator requires assigning the ASHRAE climate zone. However, for this study, the construction attributes related to exterior walls, roofs, and windows were deliberately replaced with the materials stipulated in the local standard NOM-020-ENER. Consequently, the preloaded climate zone template had no impact on the energy and building dynamics observed in the simulations.

Therefore, based on data from the ENCEVI survey, adaptations were made to the EnergyPlus™ 23.1.0 [28] simulation template. These modifications included the exclusion of thermal insulation in building materials. Furthermore, the characteristics of the household envelope were configured by the stipulations outlined in NOM-020-ENER-2011, titled “Energy Efficiency in Buildings: Building envelope for residential use.”

**Figure 8:**
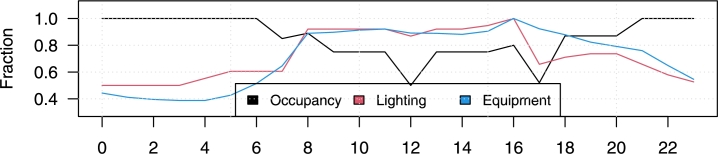
Hourly data of occupancy, lighting and equipment usage considered in the simulations.

Table 3 details the thermophysical properties of building materials according to the NOM-020-ENER standard, tailored for the three ASHRAE climate zones. It includes the window-wall ratio, which specifies the square meterage of each house facade, as well as the conditioned area and energy loads for each room. The hourly data shown in Fig. 8 clarify the relationship between occupancy, equipment use, and lighting in these environments. The occupancy levels denote the presence of two individuals in the household, peaking during hours typical of daily routines. In addition, an occupant participates in home-office activities, influencing the different patterns of equipment and lighting usage.

**Table 3:** Thermo-physical properties (construction set) employed in all the cases considering the NOM-020 ENER properties. Window-wall ratio characteristics and energy program by room.

	Material	Roughness	Thickness	Conductivity	Density	Specific heat	Thermal	Solar	U-Value	Overall U-Value
			(m)	(W/m-K)	(kg/m^3^)	(J/kg-K)	absorptance	absorptance	(W/m^2^K)	(W/m^2^K)

**Exterior Wall**	Exterior Convection	Opaque Material No Mass							13	2.009421
	Exterior Lime Mortar	Medium Smooth	0.01	0.872	1125	1000	0.9	0.7	87.2	
	HW Concrete Block	Medium Smooth	0.12	1.11	2240	900	0.9	0.8	9.25	
	Interior Lime Mortar	Medium Smooth	0.01	0.872	1125	1000	0.9	0.7	87.2	
	Interior Convection	Opaque Material No Mass							8.1	

**Interior Wall**	Generic Gypsum Board	Medium Smooth	0.0127	0.16	800	1090	0.9	0.5	12.60	2.116724
	Generic Wall Air Gap	Smooth	0.1	0.667	1.28	1000	0.9	0.7	6.67	
	Generic Gypsum Board	Medium Smooth	0.0127	0.16	800	1090	0.9	0.5	12.60	
	Exterior Convection	Opaque Material No Mass							13	

**Exterior Roof**	Reinforce Concrete Slab	Medium Rough	0.15	1.74	2322	831.5	0.9	0.7	11.6	2.006197
	Exterior Lime Mortar	Medium Smooth	0.01	0.872	1125	1000	0.9	0.7	87.2	
	Interior Convection	Opaque Material No Mass							6.6	

**Window**	Outdoor Convection	Opaque Material No Mass							13	4.838753
	Clear Glass	-	0.006	1.1	Solar Heat Gain Coefficient (SHGC): 0.6				4.87	
	Interior Convection	Opaque Material No Mass							8.1	

		North (315 to 45 deg)		East (45 to 135 deg)		South (135 to 225 deg)		West (225 to 315 deg)		Total
	Gross Wall Area (m^2^)	41.75		16.25		41.75		16.25		116
**Window-Wall**	Above Ground Wall Area (m^2^)	41.75		16.25		41.75		16.25		116
**Ratio**	Window Opening Area (m^2^)	0		0		0		6.34		6.34
	Gross Window-Wall Ratio (%)	0		0		0		39		5.46
	Above Ground Window-Wall Ratio (%)	0		0		0		39		5.46

Zone Name		Total Area		Conditioned Area		Lighting		People		Equipment
				Setpoint - Htg: 21.7 & Clg: 24.4						
		(m2)		(m^2^)		(W/m^2^)		m^2^ per person		(W/m^2^)
	Bathroom	6		0		4.4132		3		0
	Kitchen	10.5		10.5		7.9653		5.25		6.67
	Living room	42.2		42.2		9.3646		21.1		6.67
	Room 1	10.5		10.5		9.3646		5.25		6.67

Furthermore, for energy simulation, the HVAC system and its associated set points were considered to operate under the same conditions with or without shading. The shadings considered the adjustment of the shades based on (2) depending on the location and the climate characteristics of the weather file.

### 3.4 Numerical results validation

ASHRAE Guideline 14-2014 uses simplified methods to quantify uncertainty in energy modeling. The Guideline explains it as a “process of determining the degree of confidence in the true value when using measurement procedures and/or calculations” [51].

Two principal uncertainty indices are usually considered for calibrated simulations: the normalized mean bias error (NMBE) and the coefficient of variation of the root mean square error (CV(RMSE)). The NMBE, detailed in (3), should be within ±5% to be acceptable. Similarly, the CV(RMSE), described in (4), should not exceed ±15% [52].

(3) $$\text{NMBE}=\frac{1}{\overset{¯}{m}}\cdot \frac{\sum_{i=1}^{n}(m_{i}-s_{i})}{n-p}\times 100\text{\%}$$

(4) $$\text{CV(RMSE)}=\frac{1}{\overset{¯}{m}}\sqrt{\frac{\sum_{i=1}^{n}{\left(m_{i}-s_{i}\right)}^{2}}{n-p}}\times 100\text{\%}$$

where p=1, $\overset{¯}{m}$ is the arithmetic mean of the sample of *n* observations, $m_{i}$ is the measured values, and $s_{i}$ the simulation-predicted data.

The proposed household model was calibrated against an actual one-level house in Mexico City with characteristics similar to those discussed. Energy consumption was used as the validation parameter since historical information can be recovered from the electricity bill (every two months). In addition, there were also two inhabitants in the house during 2022. The measured data from this bill indicated an annual consumption of 2,089 kWh, compared to the simulated annual consumption of 2,278.97 kWh. The resulting NMBE was −0.15%, and the CV(RMSE) was 10%. The detailed calculations are shown in Table 4.

**Table 4:** Energy model calibration using ASHRAE Guideline 14-2014.

Var	12/23-02/21	02/22-04/24	04/25-06/22	06/23-08/22	08/23-10/22	10/23-12/22	Total
*n*	61	62	59	61	61	61	365
$\overset{¯}{m}$	6.13	6.03	5.98	6.20	5.49	4.51	348.17
$m_{i}$	374	374	353	378	335	275	2089
$s_{i}$	380.20	387.04	368.83	380.66	380.66	381.58	2278.97

						**NMBE**	-0.15%
						**CV(RMSE)**	10%

After initial calibration, HVAC systems were incorporated into the energy models to evaluate the impact of their use. This addition allowed for an analysis of potential changes in energy consumption in Mexico City and other locations where HVAC usage could differ significantly.

The HVAC system considered the *ideal air loads* feature in EnergyPlus™ 23.1.0 [28], [53]. This approach allows to simplify the HVAC system modeling by bypassing the complexities of specific equipment details. Ideal air loads provides a method to estimate the heating and cooling demands necessary to maintain the set point temperatures within each building zone, assuming an idealized system that can deliver the exact amount of conditioned air required. This method does not account for the dynamics of real HVAC operations, such as cycling losses or part-load performance.

This method is particularly useful in the preliminary phases of building design, where detailed HVAC specifications are not yet defined or there is no available information on the type of HVAC system. Using ideal air loads, it was sought to isolate the influence of the shading proposal in the building envelope and internal loads on overall energy requirements, thus providing a clear view of baseline heating and cooling demands independent of system efficiency.

### 3.5 Behavior of the indoor temperature

**Figure 9:**
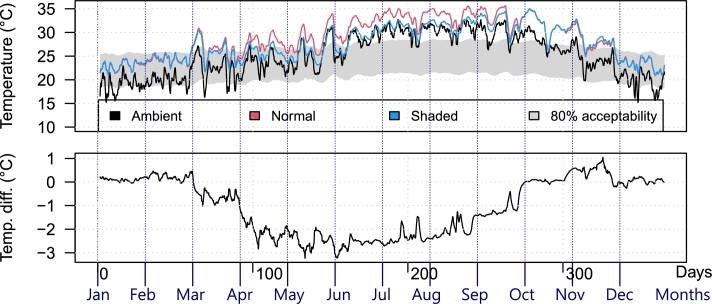
Temperature comparison example (no HVAC): the average indoor temperature of the La Paz household with and without the shading system throughout 2022.

After performing both simulations (with and without the shading system), the collected data were processed as described in Section 2.3. As anticipated, the shading system helped reduce indoor temperature only during a specific time span, depending on the shades fixed inclination and separation. Fig. 9 shows the said behavior in La Paz, without considering heating or cooling systems, where the shaded and normal indoor temperatures are almost the same during the cold season, and divert by about $2.5\;{}^{\circ }$C during the spring-summer seasons. Overall, the foreseen behavior depicted in Fig. 2 was reproduced, avoiding the sun's rays when the output temperature was high and enabling insolation to counteract the outdoor cold.

**Figure 10:**
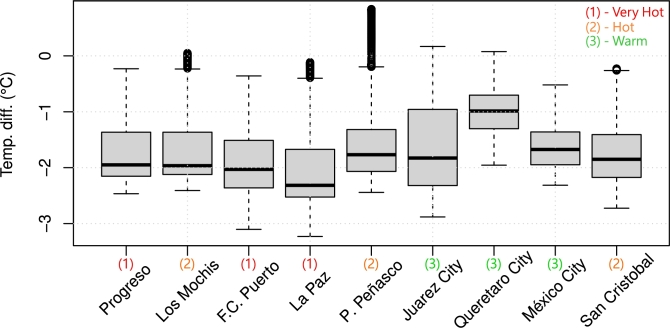
Temperature difference at every location (no HVAC).

**Figure 11:**
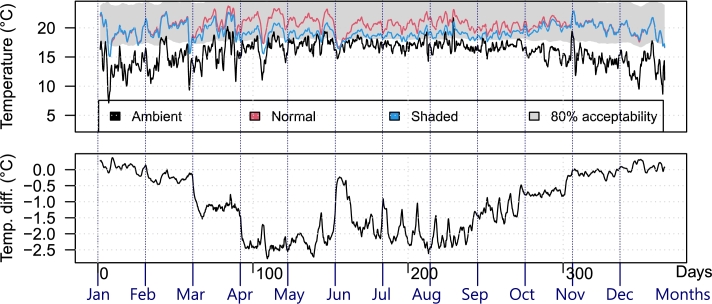
Temperature comparison example (no HVAC): San Cristobal household average indoor temperature with and without the shading system throughout 2022.

Fig. 10 summarizes the indoor temperature differences at every location during the hot season, considered between 1 May and 30 September (days 121 to 273). It can be seen that the shades reduced the indoor temperature in all locations. However, temperature reduction may not be desired in every city; for example, San Cristobal is mostly cold throughout the year (see Fig. 7) and, as shown in Fig. 11, the shading system actually harmed thermal comfort during the “hot” season. However, it can be seen that the proposed system provides the same performance even though La Paz and San Cristobal are entirely different: it affected indoor temperature only during the hot season.

### 3.6 Thermal comfort

In evaluating thermal comfort within the building, the analysis encompassed all rooms, including the bathroom and kitchen, not just the living room and Room 1, where most activities occurred. This approach was considered to demonstrate the benefits of the shading proposal across the entire house, since it was considered that the entire rooftop was covered. Table 5 shows the *80% acceptability* test from the ASHRAE Standard 55, using the adaptive thermal comfort model. It was calculated inside the simulation software using LadybugTools v1.7.0 [29]. The component used was *LB Adaptive Comfort* with a neutral offset of ($\pm 3.5\;{}^{\circ }$C) for an acceptability of 80%. For locations like La Paz, Progreso, Quintana Roo, Los Mochis, and Juarez City, there is a clear trend showing that the use of shades significantly increases the acceptability of thermal comfort in all rooms. This suggests that in these locations, the external temperatures and solar radiation levels are high enough to justify the addition of shades to help reduce indoor temperatures to more comfortable ones. Interestingly, for San Cristobal, Mexico City, and Queretaro City, shades decreased the acceptability of thermal comfort in most rooms. This indicates that existing climatic conditions already provided higher thermal comfort without additional shading systems.

**Table 5:** Thermal comfort (%) considering 80% *acceptability* from the ASHRAE Standard 55.

Location	Bathroom		Kitchen		Living Room		Room 1		Average	
	Normal	Shades	Normal	Shades	Normal	Shades	Normal	Shades	Normal	Shades
**La Paz**	56.59	70.91	49.82	69.66	44.34	61.58	53.23	69.77	51.00	67.98
**Progreso**	67.35	93.9	49.49	84.76	40.16	70.66	61.86	88.98	54.72	84.58
**F.C. Puerto**	69.97	92.18	55.53	87.03	45.82	75.02	62.67	88.82	58.50	85.76
**Los Mochis**	52.63	69.73	50.51	70.56	51.39	65.88	52.36	69.61	51.72	68.95
**Puerto Peñasco**	41.19	51.86	41.23	55.81	48.32	59.55	41.62	53.36	43.09	55.15
**San Cristobal**	46.21	13.9	74.6	33.4	84.85	51.99	59.38	24.71	66.26	31.00
**Juarez City**	35.81	44.37	36.21	47.68	40.79	50.79	35.92	46.07	37.18	47.23
**Mexico City**	42.57	25.24	61.62	43.64	72.12	58.41	48.16	34.19	56.12	40.37
**Queretaro**	45.16	36.62	60.59	51.56	69.78	61.2	51.14	44.14	56.67	48.38

La Paz, Progreso, and Felipe Carillo Puerto (ASHRAE climate zone 1) showed a significant increase in thermal comfort with rooftop shades. For example, Room 1 in Progreso increased comfort from 61.86% to 88.98%; in the kitchen, there was an increase of 35.27 percentage points (a relative increase of 71.3%). The high percentages of acceptability suggest that rooftop shading is an effective measure to counteract the effects of intense solar radiation.

For ASHRAE climate zone 2, different results were found. For example, in Los Mochis and Puerto Peñasco, the benefits of the shades were not as decisive as in very hot climates. However, the shades increased thermal comfort by at least 10.67%. At Juarez City, in ASHRAE zone 3, the household increased its thermal comfort in the range 8.56% to 11.47%.

However, in locations such as San Cristobal, Mexico City and Queretaro, the impact of rooftop shades was negative, indicating that solar gains contribute significantly to indoor comfort. The most significant decrease in comfort with rooftop shades was seen in San Cristobal. In fact, the outdoor temperatures at these locations exhibited higher HDH than CDH (see Table 2). This means that the natural need for heating exceeds the need for cooling. Since the shading system contributes to the need for cooling, the use of shades in these regions is counterproductive, especially if heating is required. Decreased indoor temperatures due to shades could increase HDH, leading to a higher demand for artificial heating to reach comfortable levels.

### 3.7 Energy savings

**Figure 12:**
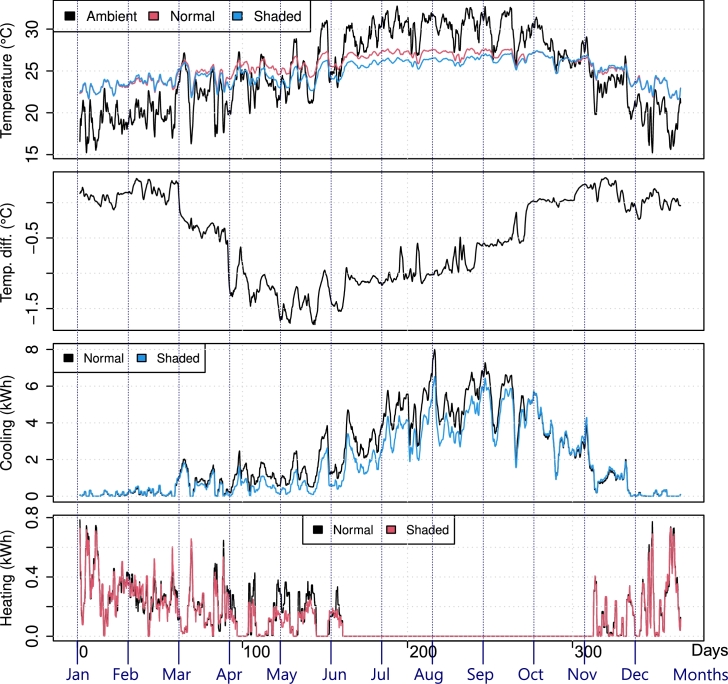
Temperature and energy consumption in La Paz, with and without the shading system throughout 2022, considering HVAC.

Now, assuming that each household has active cooling and heating systems, the shades were expected to reduce the energy consumption of the cooling operation by passively reducing indoor temperature, helping the HVAC system. In fact, the indoor temperature was kept artificially between $21.7\;{}^{\circ }$C and $24.4\;{}^{\circ }$C throughout the year. For comparison, Fig. 12 shows the results of La Paz considering active temperature control (one should recall that the shown temperature is a weighted sum of all rooms and that the bathroom was not equipped with cooling or heating systems). A temperature difference, not as big as before, was still present and in accordance with the rationale discussed previously. However, the energy needed to cool the household was effectively reduced during the hot season, and even some contingent savings in heating were found, mostly due to the simulation variability discussed.

**Figure 13:**
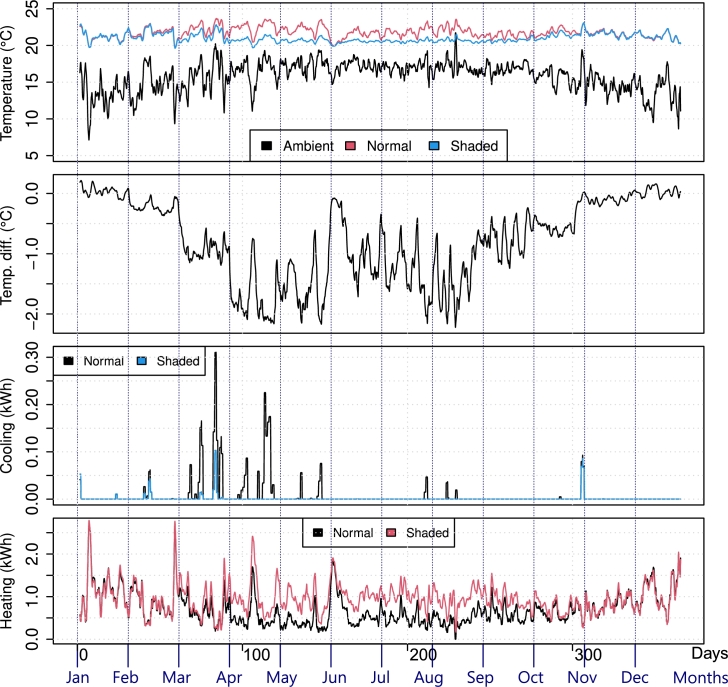
Temperature and energy consumption in San Cristobal, with and without the shading system in 2022, considering HVAC.

Similarly, Fig. 13 shows the temperature and energy consumption comparison for the San Cristobal household. Due to the previously reported results, one could expect the opposite effect in terms of energy savings. Indeed, more heating is required if the household is shaded; however, it is also noticeable that the cooling needs were mostly eliminated.

**Figure 14:**
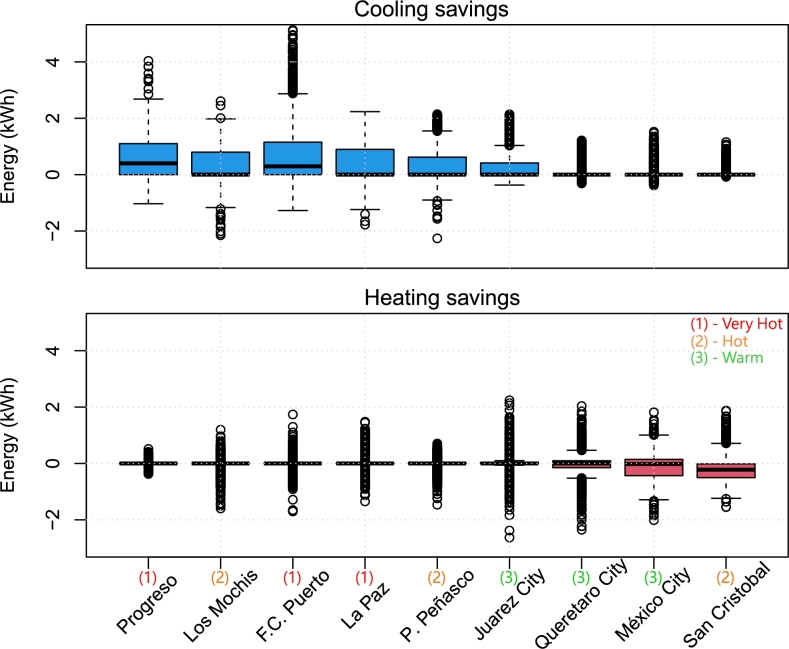
Cooling and heating energy savings hourly statistics throughout 2022 for the shaded scenario. Blue boxes show energy savings, whereas red boxes show energy losses.

The results comparing energy savings for cooling and heating throughout the year are summarized in Fig. 14, which shows the hourly statistics through box plots. There were consistent savings throughout the year for cooling and, for heating, locations in hot and very hot zones reported no increase in heating consumption (since the shades do not obstruct sunlight during the cold season), but increased heating requirements in Queretaro City, Mexico City, and San Cristobal. In fact, the shading system operated as expected during the hot season (May through September), decreasing the indoor temperature of an already “cold” environment, resulting in heating needs such as those shown in Fig. 13. These locations are consistently colder than the heating setting of $21.7\;{}^{\circ }$C throughout the year, as visible in Fig. 7.

**Figure 15:**
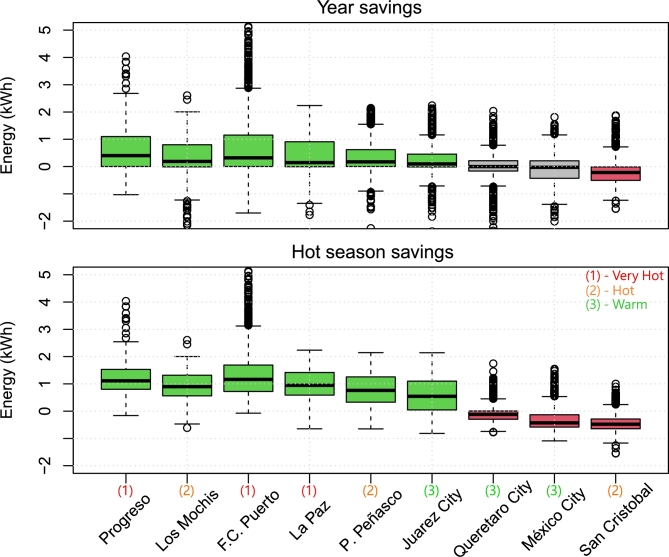
Overall energy savings hourly statistics throughout 2022: full year and hot season comparison. Green boxes indicate energy savings, red boxes indicate losses, and gray boxes show no clear changes.

The cooling and heating savings were summed throughout the year and during the hot season (May through September). The resulting hourly statistics are shown as box plots in Fig. 15. Again, locations with hot weather benefited greatly from the shading system, whereas colder locations exhibited results close to zero, and, specifically, San Cristobal reported overall energy losses. Table 6 provides a comprehensive overview of the net impact of shading on total energy savings for heating and cooling in various cities in Mexico.

**Table 6:** Summary of energy savings throughout 2022.

City	Climate	Mexico	Total heating	Total cooling	Total	Total
	zone	region	(kWh)	(kWh)	(kWh/m^2^)	(MWh)
**La Paz**	1 Very Hot	Extreme hot	71.5	3836.9	56.64	3.91
**Progreso**	1 Very Hot	Tropical	4.6	5015.0	72.75	5.02
**F.C. Puerto**	1 Very Hot	Tropical	10.2	5438.5	78.97	5.45
**Los Mochis**	2 Hot	Extreme hot	132.6	3187.7	48.12	3.32
**P. Peñasco**	2 Hot	Extreme hot	328.6	2615.2	42.66	2.94
**San Cristobal**	2 Hot	Tropical	-2207.9	57.3	-31.17	-2.15
**Juarez City**	3 Warm	Extreme hot	72.4	2359.7	35.25	2.43
**Mexico City**	3 Warm	Temperate	-712.7	182.3	-7.69	-0.53
**Queretaro City**	3 Warm	Temperate	-110.7	397.9	4.16	0.29

It was confirmed that the proposed system was effective in hot locations, reducing the required cooling energy, and having a small effect on heating requirements. In its turn, the impact in temperate locations was negligible. Finally, San Cristobal shows otherwise, with a significant increase in heating demand (2207.94 kWh), demonstrating that shades led to a substantial reduction in passive solar heating, requiring additional active heating.

The results imply that rooftop shades are an effective passive strategy to reduce cooling energy consumption in hot climates. However, their impact on heating energy demand varies and may require additional considerations to avoid increasing energy use for heating.

#### 3.7.1 Cooling

Since the shading proposal is intended to help reduce the indoor temperature, the cooling savings were analyzed in more depth. In addition, the ENCEVI [10] discards Queretaro City, Mexico City, and San Cristobal inhabitants as owners of HVAC systems. Therefore, the following analysis omits those cities.

The results of the simulation of cooling energy reflect that shading systems significantly reduced the cooling energy consumption (see Table 7). Even in cities with moderate or tropical climates, shading solutions effectively reduced energy demand. These findings underscore the importance of implementing passive shading systems as an energy-efficient measure to enhance comfort and reduce energy costs across different climate zones.

**Table 7:** Detailed yearly total cooling savings (kWh).

Location	Kitchen		Living Room		Room 1		Total		kWh/m^2^		Diff.	Savings
	Normal	Shades	Normal	Shades	Normal	Shades	Normal	Shades	Normal	Shades		
**La Paz**	3506.6	2677.3	3553.9	2842.7	12093.2	9796.8	19153.7	15316.8	277.59	221.98	-3836.87	20.03%
**Progreso**	4837.9	3701.2	4755.1	3865.1	16613.6	13625.1	26206.5	21191.5	379.80	307.12	-5015.04	19.14%
**F.C. Puerto**	3970.9	2811.9	3939.7	2943.0	13842.2	10559.6	21752.9	16314.5	315.26	236.44	-5438.45	25.00%
**Los Mochis**	3487.3	2782.2	3586.5	2985.3	11938.3	10056.9	19012.1	15824.4	275.54	229.34	-3187.69	16.77%
**P. Peñasco**	3053.0	2447.1	3101.5	2613.8	10239.2	8717.6	16393.7	13778.5	237.59	199.69	-2615.17	15.95%
**Juarez City**	1412.6	933.2	1413.6	972.4	4940.5	3501.4	7766.7	5407.0	112.56	78.36	-2359.71	30.38%

For example, in the ASHRAE climate zone 1 (very hot), La Paz had notable savings of 20.03% of cooling energy, Progreso had 19.14% of savings, and Felipe Carillo Puerto held the highest with 25% among very hot climates. In ASHRAE climate zone 2 (hot), there were also savings. In Los Mochis, the proposal saved 16.77% of energy, whereas in Puerto Peñasco it saved 15.95%.

To provide an interesting insight into the benefits of the proposed passive system, its associated energy savings were compared against a residential photovoltaic (PV) installation. Each location was tested in the *PVWatts Calculator* from the National Renewable Energy Laboratory (NREL) of the Department of Energy of the United States [54], and the installed capacity of a PV system was calculated to match the reported savings. In this way, the passive shading system can be compared with modern active technology.

The PVWatts Calculator takes into account location-specific weather variables and irradiance, as well as average efficiencies and losses of common photovoltaic (PV) systems. The photovoltaic system is assumed to be modern and new, has not yet degraded by aging, is properly maintained, and is clean. Table 8 shows the results, where the installed capacity is reported under the “Req. PV” column, the equivalent surface was calculated considering an average 6.25 m^2^/kW and reported under “Surface,” and the number of panels was calculated using an area of 1.7 m^2^ per residential panel and rounding up. Cities where the proposed system was equivalent to a larger number of solar panels are those under the “very hot” (ASHRAE) and “tropical” (Mexican classification) classifications. In fact, these cities are coastal cities.

**Table 8:** Estimated PV system sizing to match the reported energy savings.

City	Energy	Req. PV	Surface	PV panels
	(kWh)	(kW)	(m^2^)	
**La Paz**	3836.87	2.15	13.44	8
**Progreso**	5015.04	2.95	18.44	11
**F.C. Puerto**	5438.45	3.70	23.13	14
**Los Mochis**	3187.69	1.80	11.25	7
**P. Peñasco**	2615.17	1.40	8.75	6
**Juarez City**	2359.71	1.30	8.13	5

## 4 Discussion

The results align with the assertion that passive measures, particularly shading, can lead to marked reductions in the energy required for cooling, especially in hot to very hot regions, offering a sustainable solution to the increasing temperatures caused by climate change [9]. They also agree with the views of Covuelas [8], who advocate for enhanced building performance with minimal energy use by taking advantage of the site's specific environmental and constructional characteristics.

However, the increase in heating energy requirements in locations such as San Cristobal due to shading points to the complexity of applying passive strategies. It suggests that shading may require careful analysis to prevent a counterproductive increase in heating demand, especially in temperate climates, where passive solar gains are crucial to warmth [17], [18]. The results observed in temperate zones underscore the conclusions drawn by researchers who advocate adaptive or scheduled shading to maximize its benefits [9], [16], [20].

In very hot climates such as La Paz, Progreso, and F.C. Puerto, implementing rooftop shades significantly increases thermal comfort acceptability. For instance, Progreso's Room 1 shows a remarkable relative increase of 71.3% (27.12 precentage points). The results for hot climates, including Los Mochis and Puerto Peñasco, reveal a consistent pattern in which the introduction of rooftop shades contributes to energy savings and increased thermal comfort. This confirms the foreseeable correlation between the intensity of solar radiation, CDH, and the effectiveness of shading devices.

This pattern echoes the findings of García-Solórzano et al. [11], who observed a reduction in average temperatures by 0.9 ^∘^C in ASHRAE climate zone 2 by seasonally adjusting the inclinations of the blinds. Extending this research, for the purpose of this discussion, a new simulation was performed to compare the average temperature reduction. Thus, a new test was carried out in Colima using (2), with a fixed shading setup, a slope of 65.3^∘^, and a separation of 0.08 m throughout the year. This approach achieved an average temperature reduction of 1.13 ^∘^C, aligning with García-Solórzano's results [11], albeit with a simplified shading approach that does not require seasonal adjustment. In addition, there is an improvement in thermal comfort of more than 10%.

The literature suggests that through passive systems, cooling load reductions can range from 27.5% to 64.5% in various contexts [16], [6]. In this case, we found cooling savings from 16% to 30%, demonstrating that shading requires case-specific analyzes as suggested by [18], [17].

The ASHRAE Standard 55 acknowledges that individuals can adapt to different temperature ranges over time [38]. Hence, while the suggested approach may not have complete control over indoor temperature, it can significantly enhance thermal comfort. The high acceptance rates for thermal comfort suggest that these adaptations are effective for most occupants. This is particularly relevant in regions where the reliance on HVAC systems is reduced and passive strategies become paramount in achieving energy efficiency [40].

In warm temperate climates, as seen in Juarez City, Mexico City, and Queretaro City, the impact of rooftop shades is mixed. These regions exhibit moderate increases or even decreases in comfort acceptability when using shades. The results imply that shades can effectively reduce the consumption of cooling energy, but their impact on heating energy is more complex. These findings prompt a reevaluation of the “one-size-fits-all” approach in passive design. Furthermore, passive design interventions may not always lead to significant energy savings, particularly in tropical regions.

It is also important to note that these results depend on the specific parameters used in (1), which, in this case, were obtained from the hottest and coldest days of the year. However, a better fitting strategy could be followed to increase the reported benefits. For example, in [55], the authors employed a multi-objective optimization approach using a genetic algorithm to minimize both the total energy consumption and the predicted percentage of dissatisfied (PPD) by fine-tuning design parameters such as the angle, depth, and number of light shelves. Such strategies suggest a potential for adapting the optimization criteria to different climatic conditions or specific thermal comfort needs. Manjarrez, et al. [5] employed algorithms such as MOHS and NSGA-II, for cost-effective energy refurbishment scenarios at the district level. This approach allowed a dynamic and effective way to plan urban energy renovations, with the aim of significant reductions in the global warming potential while maintaining economic feasibility. In addition, other studies suggest the incorporation of economic incentives, multi-objective optimization techniques, and the balance of thermal comfort with financial costs that must be considered for future work [4], [3]. These findings imply that passive design is not a universal solution; instead, it requires customization to address the unique climatic and cultural contexts of each region [6], [8].

Furthermore, it is important to note that the shading system could be approximately as effective as the residential PV systems on the roof, ranging between an installed capacity of 2 and 4 kW in coastal regions (see Table 8). However, it is reasonable to assume that shading is much less complex than active electrical installation, requires less maintenance, is cheaper, and could be assembled with recycled materials. Although a deeper analysis and comparison would be needed to support such a statement, the results seem promising in this regard.

## 5 Conclusions

This study presents the design, adjustment, and testing of a novel static rooftop shading system as a passive alternative to indoor temperature regulation. The findings verify that the system is effective in maintaining a comfortable temperature throughout the year, for instance, in very hot zones like Progreso, the system reduces discomfort from 45.29% to 15.43%, potentially negating the necessity for supplementary heating or cooling systems. In other words, in Progreso, by using shades, the thermal comfort acceptability was improved from 54.72% to 84.58%.

Rigorous energy simulations in various Mexican climate zones have validated its effectiveness and key contributions. In particular, in buildings without active cooling, the system achieves an impressive increase in thermal comfort of up to 85.76%, meeting the acceptability threshold of 80%. For buildings with active cooling systems, the implementation of this shading solution offers substantial annual energy savings of up to 30.38% (equivalent to above 5 MWh in a 69 m^2^ household), contributing to both the cost reduction and sustainability goals. Although the system appears to be most effective in hot zones, particularly coastal cities, its performance exhibits a degree of variability depending on local factors such as ambient temperature and user behavior.

The effectiveness of rooftop shading systems varies in the ASHRAE climate zone 3. For example, in Queretaro, the implementation of a shading system resulted in a 9% reduction in thermal comfort. Despite the fact that the shades system effectively reduces cooling energy consumption by 397.9 kWh, their impact on heating energy consumption was more nuanced, showing a decrease of 110.7 kWh, reflecting a general energy savings of 4.16 kWh/m^2^. In contrast, Juarez City had an energy savings of 35.25 kWh/m^2^ and increased thermal comfort by 10%.

Studies indicate that passive systems can achieve cooling load reductions of 27.5% in different scenarios. In our investigation, cooling savings of 16% to 30% were observed. This observation highlights the importance of further research to optimize its applicability in a broader range of climate conditions, ensuring its potential for widespread adoption in various building contexts.

In contrast to other approaches advocating automated or season-specific systems, our solution provides consistent benefits year round without requiring user intervention by increasing thermal comfort by more than 15% in zones such as Zone 2, similar to season-specific system studies from other approaches.

Furthermore, it should be noted that the shading system appears to be nearly as effective as residential PV systems, which typically have an installed capacity of 2 to 4 kW in coastal areas. Importantly, shading solutions are generally less complex than active electrical installations. They require minimal maintenance, are less costly, and can even be constructed using recycled materials. The initial results are quite encouraging; however, future work should include a more detailed analysis and comparison, necessary to substantiate these observations.

Future work includes expanding the research in other countries around the world, testing the system in experimental settings, and employing artificial intelligence techniques to determine the adjustments of the shading system to reduce energy consumption during the year even further. Furthermore, when this system is applied on the roof, surveys of occupant satisfaction and thermal evaluation using thermal comfort surveys would be necessary to understand the benefits of this system from the user's perspective.

## CRediT authorship contribution statement

**Juana Isabel Méndez:** Writing – original draft, Validation, Software, Investigation, Formal analysis, Data curation. **Luis Ibarra:** Writing – review & editing, Methodology, Formal analysis, Conceptualization. **Pedro Ponce:** Visualization, Validation, Methodology, Investigation. **Alan Meier:** Writing – review & editing, Visualization, Validation, Supervision. **Arturo Molina:** Visualization, Validation, Supervision, Resources, Project administration.

## Declaration of Competing Interest

The authors declare that they have no known competing financial interests or personal relationships that could have appeared to influence the work reported in this paper.

## Footnotes

1 According to ASHRAE, there are three climate zones in Mexico (very hot, hot, and warm), or three climate regions according to the local classification (extremely hot, temperate, and tropical) [10].
